# An evaluation of screening for lung cancer in Niigata Prefecture, Japan: a population-based case–control study

**DOI:** 10.1054/bjoc.2001.2060

**Published:** 2001-09-01

**Authors:** H Tsukada, Y Kurita, A Yokoyama, S Wakai, T Nakayama, M Sagawa, H Misawa

**Affiliations:** Department of Internal Medicine, Niigata Cancer Center Hospital, Niigata, Japan; Association for Prevention of Cancer & Cardiovascular Diseases of Niigata, Niigata, Japan; Division of Epidemiology, Department of Field Research, Osaka Medical Center for Cancer and Cardiovascular Diseases, Osaka, Japan; Department of Thoracic Surgery, Institute of Development Aging and Cancer, Tohoku University, Sendai, Japan; Niigata Health & Hygiene Center, Niigata, Japan

**Keywords:** lung cancer, screening, case–control study, efficacy

## Abstract

Although an annual screening programme for lung cancer has been carried out widely in Japan since 1987, there is insufficient evidence to confirm its efficacy in terms of reducing mortality. In order to evaluate the efficacy of the lung cancer screening which has been widely carried out in Japan since 1987, a case–control study was conducted in Niigata Prefecture, Japan. In the study area, chest X-ray examinations for all participants and sputum cytology for high-risk participants were offered annually. Case subjects, who had died from lung cancer (174), and control subjects matched by sex, year of birth, residence and smoking status (801), who had been alive at the time of diagnosis of the corresponding case, were selected from the National Health Insurance holders. Screening histories of the subjects were compared between cases and matched controls for the identical calendar period before the time of diagnosis of the cases. The odds ratio of death from lung cancer for those screened within 12 months vs those not screened was 0.401 (95% CI: 0.272–0.591) with adjustment by smoking index. Our results suggest that annual lung cancer screening might reduce mortality from lung cancer by approximately 60%. © 2001 Cancer Research Campaign


British Journal of Cancer (2001) 85(9), 1326–1331
© 2001 Cancer Research Campaign
doi: 10.1054/ bjoc.2001.2060, available online at http://www.idealibrary.com on IDEAL
http://www.bjcancer.com

### An evaluation of screening for lung cancer in Niigata Prefecture, Japan: a population-based case–control study

**H Tsukada[1], Y Kurita[1], A Yokoyama[1], S Wakai[2], T Nakayama[3], M Sagawa[4] and H Misawa[5]**

[1]Department of Internal Medicine, Niigata Cancer Center Hospital, Niigata, Japan; [2]Association for Prevention of Cancer & Cardiovascular Diseases of Niigata, Niigata, Japan; [3]Division of Epidemiology, Department of Field Research, Osaka Medical Center for Cancer and Cardiovascular Diseases, Osaka, Japan; [4]Department of Thoracic Surgery, Institute of Development, Aging and Cancer, Tohoku University, Sendai, Japan; [5]Niigata Health & Hygiene Center, Niigata, Japan

**Summary** Although an annual screening programme for lung cancer has been carried out widely in Japan since 1987, there is insufficient evidence to confirm its efficacy in terms of reducing mortality. In order to evaluate the efficacy of the lung cancer screening which has been widely carried out in Japan since 1987, a case–control study was conducted in Niigata Prefecture, Japan. In the study area, chest X-ray examinations for all participants and sputum cytology for high-risk participants were offered annually. Case subjects, who had died from lung cancer (174), and control subjects matched by sex, year of birth, residence and smoking status (801), who had been alive at the time of diagnosis of the corresponding case, were selected from the National Health Insurance holders. Screening histories of the subjects were compared between cases and matched controls for the identical calendar period before the time of diagnosis of the cases. The odds ratio of death from lung cancer for those screened within 12 months vs those not screened was 0.401 (95% CI: 0.272–0.591) with adjustment by smoking index. Our results suggest that annual lung cancer screening might reduce mortality from lung cancer by approximately 60%. © 2001 Cancer Research Campaign

**Keywords**: lung cancer; screening; case–control study; efficacy

The incidence and mortality of lung cancer have been increasing in Japan and lung cancer is the leading cause of cancer death. An attempt to reduce lung cancer mortality is an issue of great importance. Because cigarette smoking is the predominant cause, lung cancer is mainly preventable. However, anti-smoking activities need a latent period before a substantial reduction of mortality becomes apparent. Since long-term smokers remain at high risk for prolonged periods even after quitting, effective secondary prevention measures are also important.

In Japan since 1987 local governments have been obligated to offer an annual screening programme for lung cancer using chest X-ray and/or sputum cytology, to residents aged 40 or over, under the Health and Medical Services Law for the Aged. However, so far there is insufficient evidence to confirm its efficacy in terms of reducing mortality. It is necessary to assess whether this screening programme is effective or not from the standpoint of public health policies.

A randomized controlled trial is the best approach to evaluate efficacy of the screening. Although several randomized trials in the United States and in Europe have been conducted to evaluate lung cancer screening, none of them showed any evidence of benefit in terms of mortality reduction (Fontana et al, 1986; Tockman, 1986; Melamed and Flehinger, 1987; Kubik et al, 1990). In Japan, a randomized controlled trial would be difficult to conduct, because annual chest X-ray screening is already widespread under the Tuberculosis Control Law since the 1950s.

The case–control study is an alternative approach to examine the efficacy of screening. Although there are several potential biases (Hosek et al, 1996; Cronin et al, 1998), this approach can be applied in situations where a screening test has been widespread in the general public before its efficacy has been determined by clinical trials.

In this paper, we reported the results of a case–control study evaluating lung cancer screening in Niigata Prefecture, Japan.

### SUBJECTS AND METHODS

### Study setting

In Niigata Prefecture, Japan, screening for lung cancer using chest X-ray and sputum cytology has been performed since 1985. The records of screening participants, i.e., name, date of birth, address and results of screening test, are stored in each municipality. From 1990 to 1997, a total of 243 785 people participated in the screening, and 1721 cases of lung cancer were detected. After screening by chest X-ray and/or sputum cytology, 1.0–2.8% of all participants were referred to hospitals for diagnostic and therapeutic procedures every year, and 95–100% of referred people actually visited hospitals (Yokoyama, 1999). In 12 municipalities of this study area from 1995 to 1998, the rate of clinical stage I disease for cases detected by screening was 76%, and was 24% for symptomatic cases. In addition, the 5-year survival rates were 51% and 15%, respectively (Tsukada et al, 2000).

Received 18 January 2001
Revised 9 July 2001
Accepted 9 July 2001

Correspondence to: H Tsukada

### Source population

The study areas consisted of 17 municipalities of relatively small populations (the populations aged 40 or over were less than 15 000), mostly in rural areas where the populations of middle-aged or elderly people were stable. Data during 1990–1997 in 14 municipalities and 1992–1997 in 3 municipalities were collected and examined in this study. We defined the source population as National Health Insurance holders in the study area, aged 40 or over (47 117 people). All were invited to lung cancer screening annually under the Health and Medical Services Law for the Aged.

### Screening

Both chest X-ray for all participants and sputum cytology for high-risk group were used for screening. Chest X-rays were taken by miniature photofluorography with 140 kv, using 100 × 100 mm film. Saccomanno's 3-day pooled method was used for sputum cytology. The high-risk group for sputum cytology was defined as those who ranked 400 or more on the smoking index (average number of cigarettes smoked per day multiplied by the number of years smoked). Those who were suspected of having lung cancer were referred to hospitals.

### Identification of cases

In 1990–97, all deaths due to lung cancer in the source populations were identified by death certificates and/or through the Niigata Cancer Registry. Case subjects were defined as the people who (1) had been National Health Insurance holders, (2) had died of lung cancer between the ages of 40 and 79, (3) had been diagnosed during the study period, (4) had lived in the same area since the beginning of the study period.

In order to increase the efficiency of identifying controls matched by smoking habits, cases were limited to the high-risk group for males and the non-high-risk group for females, because of the high smoking prevalence in males and low smoking prevalence in females in Japan.

Information at diagnosis was collected from the medical records of the hospitals where the case subjects were diagnosed as lung cancer. Clinical stage was determined according to UICC staging system (UICC, 1992). Migration status was investigated from records of residence obtained from local governments. Information on smoking habits for the cases were obtained from medical records, screening records and interview surveys. When there were discrepancies in information, priority was given to the higher smoking index.

A total of 580 deaths due to lung cancer were identified in the source population during the study period. Of these, 162 cases were excluded because they had been either over 79 years old or under 40 years old at the time of death. In 7 cases, through the review of medical records, their deaths turned out to be due to other causes. 129 cases were found to have been non-National Health Insurance holders and 26 cases had been diagnosed as having lung cancer before the study period. In addition, 22 cases were excluded because they had moved into the municipality during the study period. 15 cases in males were excluded because they were classified in the non-high-risk group and 9 female cases in the high-risk group. Smoking habits of 6 cases could not be determined. In 24 other cases, the date of diagnosis could not be identified, because of destruction or loss of medical records (3 cases) or physician's refusal (3 cases). As a result, a total of 180 cases were collected.

For cases covered by the above process, an additional interview survey was conducted with the families to investigate not only smoking habits, but also some confounding factors, namely, (i) frequency of health check-ups other than the lung cancer screening and (ii) routine consultations at local medical facilities, both of which appeared to be associated with the opportunities of examination by chest X-ray. Well-trained public health nurses who belonged to local health centres or local governments conducted these interviews, and they visited the houses of cases or phoned and used a uniform questionnaire.

### Identification of controls

For each case an attempt was made to select 10 controls from the list of National Health Insurance holders at the beginning of the study period. In 9 municipalities, the list at the beginning was not available, and the list was reconstructed from the oldest available list and the file of death certificates before the list was made. In each study area, all cases were identified in the list. To ensure that case and control subjects had equal access to screening during the exposure period, control candidates were selected from those located near the case, matched by sex and year of birth (±2 years). In addition, controls were required to have been alive and National Health Insurance holders at the time when the corresponding case was diagnosed. As a result, 1770 control candidates were selected.

Controls were interviewed, using the same questionnaire as cases, by visiting and/or telephone in 16 areas and mail alone in one area. If deceased, a family member was interviewed if possible. The smoking index for controls was calculated using the years smoked up to the time when the corresponding case was diagnosed. Interviews were continued until 5 appropriate controls (high-risk males or non-high-risk females) were obtained.

During these processes, 413 control candidates were excluded because they were classified either in the non-high-risk group for males or in the high-risk group for females. 44 control candidates were excluded because of migration into the area after the diagnosis of the matched case. In addition, 101 controls were excluded because getting information from them was impossible due to death, emigration, or refusal. In one town, the questionnaires of excluded controls were discarded immediately, and the reason why 21 candidates were excluded was unclear.

In total, 825 controls were selected from 1770 control candidates.

### Identification of screening history

Screening histories of both cases and controls were obtained from the lists of screened people kept at local government offices or local health centres. Screening histories of cases and matched controls were reviewed within the same calendar period before the case was diagnosed as lung cancer. Controls were not counted as screened if this occurred after the diagnosis of the 'case' lung cancer. In some municipalities, screening records of decedants are destroyed after an interval. Since the screening records of 6 cases within 12 months before diagnosis had been erased, these 6 cases and 24 matched controls were excluded. The records of the 12 controls which had been destroyed (though the corresponding case records were available) were regarded as unscreened in the relevant 12 months in order not to overestimate the efficacy of screening.

Since it was impossible to check for the existence of symptoms, we considered those who participated in a screening programme as

© 2001 Cancer Research Campaign 

having an actual screening test, that is, a test that leads to a diagnosis in the absence of symptoms.

Finally, 174 cases and 801 controls were used for the analysis.

### Analyses

The odds ratio of dying from lung cancer for screened vs non-screened people and 95% confidence interval (CI) were calculated using conditional logistic regression analysis with PHREG Procedure in the SAS computer program. Smoking-adjusted odds ratios were calculated by including 8 dichotomous variables on the smoking index (200 to 399, 400 to 599, 600 to 799, 800 to 999, 1000 to 1199, 1200 to 1399, 1400 to 1599 and 1600 or more) in the model. Screening histories were categorized as dichotomous whether the study subjects had been screened or not within the 0–12 and 12–24 months before the case was diagnosed as lung cancer.

Information about other health check-ups and the frequency of routine medical consultation were obtained from the interview surveys for 152 cases and 678 controls. In order to estimate the confounding effects, these variables were included in the model, according to the methods described by Sobue et al (1992).

Sub-group analyses of the odds ratios according to sex, age, histologic type of tumour were also performed with conditional logistic regression model, holding each matched set.

**Table 1** Characteristics of the cases

| | Males | | Females | |
|---|---|---|---|---|
| | *n* | % | *n* | % |
| Age | | | | |
| 40–49 | 4 | 2.7 | 1 | 4.0 |
| 50–59 | 7 | 4.7 | 4 | 16.0 |
| 60–69 | 65 | 43.6 | 6 | 24.0 |
| 70–75 | 51 | 34.2 | 8 | 32.0 |
| 76–79 | 22 | 14.8 | 6 | 24.0 |
| Histologic type | | | | |
| Squamous cell carcinoma | 47 | 31.5 | 1 | 4.0 |
| Adenocarcinoma | 57 | 38.2 | 19 | 76.0 |
| Small cell carcinoma | 32 | 21.5 | 1 | 4.0 |
| Large cell carcinoma | 1 | 0.7 | 1 | 4.0 |
| Unknown | 12 | 8.1 | 3 | 12.0 |
| Clinical stage | | | | |
| I | 18 | 12.1 | 2 | 8.0 |
| II | 5 | 3.3 | 1 | 4.0 |
| IIIA | 27 | 18.1 | 0 | 0.0 |
| IIIB | 31 | 20.8 | 10 | 40.0 |
| IV | 61 | 40.9 | 11 | 44.0 |
| Unknown | 7 | 4.7 | 1 | 4.0 |
| Total | 149 | 100.0 | 25 | 100.0 |

### RESULTS

The characteristics of deceased cases are shown in Table 1. Out of 174 cases, 159 cases (91.4%) were diagnosed with histological and/or cytological evidence of lung cancer, while the remaining 15 cases were diagnosed by chest X-ray and/or clinical findings alone. The predominant histologic type for females was adenocarcinoma, whereas squamous cell carcinoma and small cell carcinoma were the major types in male cases. Over two thirds of the cases of both sexes had stage III or stage IV cancers at diagnosis.

Table 2 shows the distribution by smoking index at diagnosis for cases and controls. Although smoking status was matched (smoking index ≥ 400), male cases had significantly higher smoking indexes than controls ($P = 0.001$).

Table 3 shows the odds ratios of dying from lung cancer among individuals with screening histories as compared with those not screened. The smoking adjusted odds ratio of dying from lung cancer for those who were screened within 12 months compared with those not screened was 0.401 (95% CI: 0.272–0.591). We also calculated the odds ratio of dying from lung cancer for those who were screened during 12 to 24 months compared with those not screened (excluding those screened within 12 months). The odds ratio was 1.418 and 95% CI was 0.634–3.173, suggesting that no reduction in risk exists over 1 year after a negative screening result (Table 3).

Table 4 shows the odds ratios when the 2 variables, which might be associated with chest X-ray examination outside the screening programme, were added to the model. Analyses were limited to 152 cases and 678 controls, for which information on these variables was available; the non-exposed group was used as the reference group. Health check ups other than lung cancer screening and routine medical consultations are not associated with reduced mortality for lung cancer. Adjustment for these two variables scarcely alters the association with lung cancer screening.

Table 5 shows the odds ratios according to age, sex and histologic type. A statistically significant reduction of the risk for lung cancer death was observed for all ages in males. Odds ratios were lower in males than in females although the 95% CIs overlapped.

**Table 2** Smoking index of cases and controls

| | Males | | | | Females | | | |
|---|---|---|---|---|---|---|---|---|
| Smoking index | Cases | | Controls | | Cases | | Controls | |
| | *n* | % | *n* | % | *n* | % | *n* | % |
| 0 | 0 | 0.0 | 0 | 0.0 | 22 | 88.0 | 119 | 98.3 |
| 1–399 | 0 | 0.0 | 0 | 0.0 | 3 | 12.0 | 2 | 1.7 |
| 400–599 | 11 | 7.4 | 160 | 23.5 | 0 | 0.0 | 0 | 0.0 |
| 600–799 | 13 | 8.7 | 146 | 21.5 | 0 | 0.0 | 0 | 0.0 |
| 800–1199 | 78 | 52.3 | 256 | 37.6 | 0 | 0.0 | 0 | 0.0 |
| 1200–1399 | 9 | 6.0 | 27 | 4.0 | 0 | 0.0 | 0 | 0.0 |
| 1400–1599 | 8 | 5.4 | 31 | 4.5 | 0 | 0.0 | 0 | 0.0 |
| 1600+ | 30 | 20.1 | 60 | 8.8 | 0 | 0.0 | 0 | 0.0 |
| Total | 149 | 100.0 | 680 | 100.0 | 25 | 100.0 | 121 | 100.0 |

 *© 2001 Cancer Research Campaign*

**Table 3** Odds ratios of dying from lung cancer for those screened vs. unscreened according to the period for comparing screening histories before diagnosis of the case

| Months before diagnosis | Number of subjects available[a] | | Number of subjects screened (%)[b] | | Smoking-adjusted odds ratio[c] (95% Confidence interval) |
|---|---|---|---|---|---|
| | Cases | Controls | Cases | Controls | |
| 0–12 | 174 | 801 | 61 (35.0) | 450 (56.2) | 0.40 (0.27–0.59) |
| 12–24[d] | 85 | 205 | 14 (16.5) | 29 (14.1) | 1.42 (0.63–3.17) |

[a]The number of subjects who had the chance to participate in screening during the period. [b]Subjects who were screened in the period/ number of subjects (%) [c]Calculated for previous screening history 0–12 and 12–24 months before case diagnosis, compared with no screening history in those intervals, using conditional logistic regression analysis. [d]Cases and controls who had been screened within 12 months before diagnosis were excluded. Cases and controls who had no matched subjects were also excluded.

**Table 4** Odds ratios of dying from lung cancer for those screened within 12 months before diagnosis in which variables associated with the chest X-ray examination were included[a]

| Variables | Exposure (%) | | Smoking-adjusted odds ratio (95% confidence interval) |
|---|---|---|---|
| | Cases | Controls | |
| Lung-cancer screening[b] | 35 | 57 | 0.420 (0.276–0.64) |
| Other health check-ups[c] | 28 | 29 | 0.855 (0.55–1.33) |
| Routine medical consultation[d] | 66 | 55 | 1.492 (0.997–2.23) |

[a]Analyses were limited to 152 cases and 678 controls for which information on additional variables were available. [b]Attendance at screening offered by local government within 12 months. [c]Attendance at any kind of health check-up except the above screening at least annually. [d]Routine consultations at least every 3 months for any reason.

**Table 5** Odds ratios of dying from lung cancer for those screened within 12 months before diagnosis by sex, age and histologic type

| | Number of | | Screened (%) | | Smoking-adjusted odds ratio (95% confidence interval) |
|---|---|---|---|---|---|
| | Cases | Controls | Cases | Controls | |
| Sex/Age | | | | | |
| Male | | | | | |
| 40–69 | 87 | 398 | 34.5 | 57.0 | 0.367 (0.209–0.645) |
| 70–79 | 62 | 282 | 33.9 | 56.7 | 0.405 (0.207–0.792) |
| Total | 149 | 680 | 34.2 | 56.9 | 0.373 (0.245–0.569) |
| Female | | | | | |
| 40–69 | 12 | 61 | 66.7 | 65.6 | 1.101 (0.248–4.885) |
| 70–79 | 13 | 60 | 15.4 | 38.3 | 0.319 (0.065–1.560) |
| Total | 25 | 121 | 40.0 | 52.1 | 0.614 (0.225–1.675) |
| Histologic type | | | | | |
| Squamous | 48 | 220 | 27.1 | 55.0 | 0.212 (0.088–0.510) |
| Adeno | 76 | 347 | 34.2 | 55.3 | 0.431 (0.240–0.773) |
| Small | 33 | 160 | 45.5 | 58.8 | 0.592 (0.248–1.410) |
| Unknown | 15 | 67 | 40.0 | 56.7 | 0.506 (0.147–1.749) |

Squamous: squamous cell carcinoma; Adeno: adenocarcinoma; Small: small cell carcinoma.

The odds ratio for squamous cell carcinoma was lower than that for adenocarcinoma although the reduction of the risk for lung cancer death was significant in both.

### DISCUSSION

In this case–control study, an approximately 60% reduction of the risk for lung cancer death was observed for those who were screened within 12 months before the diagnosis of lung cancer, compared with those unscreened.

In a previous case–control study in Japan (Sobue et al, 1992), the odds ratio of dying from lung cancer for those screened (in 1981–88) vs non-screened was 0.72 (95% CI: 0.50–1.03). The odds ratio in our study (evaluating screening in 1990–1997) was 0.401 (95% CI: 0.272–0.591), which was statistically significant in spite of smaller sample size than the former study.

*© 2001 Cancer Research Campaign* 

During the 1980s, there was a technological advance in miniature fluorography and an improvement in quality control of screening system. At present, a 100 × 100 mm photofluorograph taken with a mirror camera, 140 kV X-ray beam and a rare earth-intensifying fluorescent screen are used in lung cancer screening. Two physicians, whose specialty is respiratory diseases, independently interpret the X-ray findings and compare the films with previous ones when any abnormalities are found. This current standard screening system became widespread in the late 1980s.

From 1995 to 1998, in 12 municipalities included in this study area, stage I diseases were detected on population-based screening 3 times as frequently as by symptoms (Tsukada et al, 2000), which suggests a higher proportion of curable cancers compared with those detected by other means. In fact, it has to be noted that the 5-year survival rate for lung cancer patients detected by screening between 1990 and 1997 was 48.1% in Niigata Prefecture (Yokoyama, 1999), which is quite different from the findings in a case–control study conducted in East Berlin that showed no beneficial evidence of lung cancer screening. The 5-year survival rate for asymptomatic lung cancer patients in the East Berlin Study was reported as 6.8% (Ebeling et al, 1987).

However, our study has several methodological problems.

(1) Although some investigators recommend only screening done in the absence of symptoms should be counted (Cole and Morrison, 1980; Cronin et al, 1998), case subjects in this study were not investigated as to whether they were symptom-derived screenees or not. The symptom-derived screening visits among the case subjects may have led to a substantial underestimation of screening efficacy.

(2) Although we tried to reduce the influence of smoking habits in controlling for self-selection bias, we did not take other confounding factors into consideration, such as environmental exposure and vegetable intake. The possibility that a more healthy lifestyle among controls could lead to over-estimation of screening efficacy cannot be ruled out.

(3) Information on the variables associated with the chances of chest X-ray outside the lung cancer screening was obtained differently from cases (family member only interviewed) and controls themselves interviewed. Furthermore, the questionnaire asked if they routinely consulted a doctor or not, or if they received health check-ups other than the lung cancer screening. These variables did not imply that they had actually had chest X-rays. Confounding could arise if lung cancer cases more commonly had chest X-ray for co-morbid disease, such as emphysema. For some of the cases, the background disease for routine medical consultation had been hypertension or diabetes, so that the chances of chest X-ray might not have been high. The data in Table 4 must therefore be interpreted with caution.

(4) 26 cases diagnosed before 1990 were excluded in the present study. To restrict cases to persons diagnosed with cancer during a given period of time and fail to include these 26 cases may produce a falsely low odds ratio and, thus, an overestimation of the efficacy of screening (Weiss and Lazovich, 1996). However, it is probable that the extent of bias from this source would be small because the excluded cases comprise only a relatively small fraction of the total cases.

(5) We had to exclude 30 case candidates. If the attendance rate for screening of these 30 case candidates (6 excluded due to unidentified smoking habits, 24 to unidentified date of diagnosis) was higher than that of 178 adopted cases, the efficacy of the screening might be overestimated. Since the date of diagnosis was unclear, attendance rate within 12 months before diagnosis was unable to be determined. However, screening histories between 1990 and the date of death were able to be identified in 23 of 30 case candidates, and only 6 of the 23 (26.1%) had been screened during the period. The attendance rate of the 23 case candidates within 12 months before diagnosis was no more than 26.1%, and the rate was lower than that of 178 adopted cases (35.0%). Accordingly, overestimation of efficacy caused by exclusion of these cases was considered to be minimum in this study.

(6) The reasons why 21 control candidates were excluded were unclear. However, 10 of 21 (47.6%) candidates were screened within 12 months before the corresponding case was diagnosed, and the attendance rate was higher than that of the cases (35.0%). The odds ratio would not be changed much even if 21 candidates were not excluded.

(7) 101 non-respondent controls were excluded from the analysis, which might cause some bias. In order to assess the possible bias, an additional analysis was performed using the screening histories of 73 of these 101 non-respondents (the other 28 non-respondents could not be obtained). Of these 73 controls, attendance rate within 12 months before diagnosis was only 16.5%, which was lower than that of cases (35.0%), so that their exclusion might overestimate the efficacy of the screening. The odds ratio was then calculated including these 73 candidates both with and without smoking-adjustment (assigning smoking index to the median of adopted controls; 830 for males and 0 for females). The odds ratio without smoking-adjustment was 0.419 (95% CI: 0.290–0.606), and with smoking-adjustment 0.436 (95% CI: 0.297–0.642). The change of the odds ratio was therefore relatively small.

To define the appropriate screening interval, screening histories were categorized whether subjects had been screened or not in the 0–12 and 12–24 months before the diagnosis of the cases. Excluding those screened within 12 months the odds ratio for those screened during 12–24 months was calculated as 1.418 and thus, no reduced risk was shown. Since the duration of detectable pre-clinical phase associated with chest X-ray examination is relatively short, lung cancer does not seem as suitable for mass-screening as uterine cervical cancer (Clarke and Anderson, 1979) and colorectal cancer (Faivre et al, 1999) for which screening once every 2 years appears to be efficacious.

In the present study, a significant protective effect of screening on mortality was not observed for females unlike the previous study in Japan (Sobue et al, 1992). It should be noted that the odds ratio for squamous cell carcinoma tended to be lower than that for adenocarcinoma, although the 95% CIs overlapped. The greater effect in males than females and also in squamous cell carcinoma than adenocarcinoma may imply that an appreciable effect might be derived more from sputum cytology than from chest X-ray in our screening programme. In the present study, any screening visit was counted as ‘screened’, regardless of the examination (X-ray + sputum/X-ray only), so that an independent effect of sputum cytology could not be evaluated and further studies are required.

Recently, the low-radiation-dose computed tomography (CT) has been shown to greatly increase the likelihood of detection of smaller, probably earlier, lung cancers than do chest X rays

 *© 2001 Cancer Research Campaign*

(Henschke et al, 1999). Our findings may point towards evaluating the efficacy of CT screening for lung cancer.

In our study, the odds ratio of dying from lung cancer for those screened within 12 months of case diagnosis vs those not screened was 0.401 (95% CI 0.272–0.591), but we could not avoid self-selection bias. Since it has probably overestimated the efficacy of the screening, the findings cannot refute the conclusions of large-scale randomized trials conducted in the 1970s and 1980s. However, our results suggest that, with the current standard unique method in Japan using miniature fluorography with independent double interpretation under good quality control, significant mortality reduction from lung cancer could be expected.

### ACKNOWLEDGEMENTS

We thank all the municipalities that participated in this study and all the staff members of Niigata Health & Hygiene Center and Association for Prevention of Cancer & Cardiovascular Diseases of Niigata for their cooperation. We also wish to thank Dr Y Nozawa, Dr H Sega, Dr H Obata, Dr Y Shimazu, Dr H Tanabe, Dr M Haraguchi, Dr K Sakurai, Dr T Saitoh, Dr T Honma, Dr Y Maruyama, Dr S Mitsuma, Dr T Tsuchiya, Dr H Terada, Dr Y Usuda, Dr E Suzuki, Dr A Iwashima, Dr K Satoh and Dr T Satoh. This study was supported by grants-in-aid for Cancer Research (9–7), from the Ministry of Health and Welfare, Japan.

### REFERENCES

Clark EA and Anderson TW (1979) Does screening by “pap” smears help prevent cervical cancer? *Lancet* **2**: 1–4

Cole P and Morrison AS (1980) Basic issues in population screening for cancer. *J Natl Cancer Inst* **64**: 1263–1272

Cronin KA, Weed DL, Connor RJ and Prorok PC (1998) Case–control studies of cancer screening: theory and practice. *J Natl Cancer Inst* **90**: 498–504

Ebeling K and Nischan P (1987) Screening for lung cancer-results from a case–control study. *Int J Cancer* **40**: 141–144

Ebeling K, Nischan P and Zellmer C (1987) Survival rates of asymptomatic or symptom-detected cases of lung cancer. *Arch Geschwulstforsch* **57**: 61–67

Faivre J, Tazi MA, Mrini TEI, Lejeune C, Benhamiche AM and Dassonville F (1999) Faecal occult blood screening and reduction of colorectal cancer mortality: a case-control study. *Br J Cancer* **79**: 680–683

Fontana RS, Sanderson DR, Woolner LB, Taylor WF, Miller WE and Muhm JR (1986) Lung cancer screening: the Mayo Program. *J Occup Med* **28**: 746–750

Henschke CI, McCauley DI, Yankelevitz DF, Naidich DP, Mcguinness G, Miettinen OS, Libby DM, Pasmantier MW, Koizumi J, Altorki NK and Smith JP (1999) Early Lung Cancer Action Project: overall design and findings from baseline screening. *Lancet* **354**: 99–105

Hosek RS, Flanders WD and Sasco AJ (1996) Bias in case-control studies of screening effectiveness. *Am J Epidemiol* **143**: 193–201

Kubik A, Parkin DM, Khlat M, Erban J, Polak J and Adamec M (1990) Lack of benefit from semi-annual screening for cancer of the lung: follow-up report of a randomized controlled trial on a population of high-risk males in Czechoslovakia. *Int J Cancer* **45**: 26–33

Melamed MR and Flehinger BJ (1987) Detection of lung cancer: highlights of the Memorial Sloan-Kettering Study in New York City. *Schweiz Med Wschr* **117**: 1457–1463

Sobue T, Suzuki T, Naruke T and The Japanese Lung-cancer-Screening Research Group (1992) A case-control study for evaluation lung-cancer screening in Japan. *Int J Cancer* **50**: 230–237

Tockman MS (1986) Survival and mortality from lung cancer in a screened population: the Johns Hopkins study. *Chest* **89**(Suppl): 324–325

Tsukada H, Yokoyama A, Kurita Y and Misawa H (2000) Evaluation of population-based lung cancer screening in Niigata and analysis of interval cases based on comparison lung cancer registry with screening records. *J Jpn Respir Soc* **38**: 501–508 (in Japanese)

UICC (1992) TNM classification of malignant tumours, 4th ed., 2nd revision, pp 77–80. Springer-Verlag: New York

Weiss NS and Lazovich D (1996) Case-control studies of screening efficacy: the use of persons newly diagnosed with cancer who later sustain an unfavorable outcome. *Am J Epidemiol* **143**: 319–322

Yokoyama A (1999) Evaluation of screening for lung cancer. In: The reports of the tasks evaluating cancer screening in Niigata Prefecture, pp 85–88. (in Japanese)

*© 2001 Cancer Research Campaign* 
